# The PduQ Enzyme Is an Alcohol Dehydrogenase Used to Recycle NAD^+^ Internally within the Pdu Microcompartment of *Salmonella enterica*

**DOI:** 10.1371/journal.pone.0047144

**Published:** 2012-10-15

**Authors:** Shouqiang Cheng, Chenguang Fan, Sharmistha Sinha, Thomas A. Bobik

**Affiliations:** 1 Department of Microbiology-Immunology, Feinberg School of Medicine, Northwestern University, Chicago, Illinois, United States of America; 2 Department of Molecular Biophysics & Biochemistry, Yale University, New Haven, Connecticut, United States of America; 3 Department of Biochemistry, Biophysics and Molecular Biology, Iowa State University, Ames, Iowa, United States of America; University of Osnabrueck, Germany

## Abstract

*Salmonella enterica* uses a bacterial microcompartment (MCP) for coenzyme B_12_-dependent 1,2-propanediol (1,2-PD) utilization (Pdu). The Pdu MCP consists of a protein shell that encapsulates enzymes and cofactors required for metabolizing 1,2-PD as a carbon and energy source. Here we show that the PduQ protein of *S. enterica* is an iron-dependent alcohol dehydrogenase used for 1,2-PD catabolism. PduQ is also demonstrated to be a new component of the Pdu MCP. In addition, a series of in vivo and in vitro studies show that a primary function of PduQ is to recycle NADH to NAD^+^ internally within the Pdu MCP in order to supply propionaldehyde dehydrogenase (PduP) with its required cofactor (NAD^+^). Genetic tests determined that a *pduQ* deletion mutant grew slower than wild-type *Salmonella* on 1,2-PD and that this phenotype was not complemented by a non-MCP associated Adh2 from *Zymomonas* that catalyzes the same reaction. This suggests that PduQ has a MCP-specific function. We also found that a *pduQ* deletion mutant had no growth defect in a genetic background having a second mutation that prevents MCP formation which further supports a MCP-specific role for PduQ. Moreover, studies with purified Pdu MCPs demonstrated that the PduQ enzyme can convert NADH to NAD^+^ to supply the PduP reaction in vitro. Cumulatively, these studies show that the PduQ enzyme is used to recycle NADH to NAD^+^ internally within the Pdu MCP. To our knowledge, this is the first report of internal recycling as a mechanism for cofactor homeostasis within a bacterial MCP.

## Introduction

Bacterial microcompartments (MCPs) are a functionally diverse group of proteinaceous subcellular organelles used to optimize metabolic pathways that have toxic or volatile intermediates [1], [2], [3], [4], [5]. They are polyhedral in shape, 100–150 nm in cross-section, and consist of metabolic enzymes encapsulated within a protein shell. The function of MCP shells is to restrict the outward diffusion of toxic/volatile metabolic intermediates and help channel them to downstream enzymes thereby minimizing toxicity or carbon loss [6], [7], [8], [9], [10]. Based on sequence analyses, MCPs are found in 15–20% of bacteria and participate in 7 or more different metabolic processes [1], [3], [4], [5], [11]. Different types of MCPs have related protein shells but differ in their encapsulated enzymes. The carboxysome, which is the archetypal MCP, is found in the majority of autotrophic bacteria where it plays a critical role in global carbon fixation [2], [5]. Other MCPs are used for the catabolism of 1,2-propanediol (1,2-PD) or ethanolamine, or have unknown functions [4], [8], [12], [13], [14]. In the enterica bacteria, 1,2-PD and ethanolamine degradation are linked to pathogenesis [15], [16], [17], [18], [19], [20]. In addition, MCPs have a number of potential biotechnology applications since they provide a foundation for the design of synthetic protein cages for use as nanoscale intracellular chemical reactors or as drug delivery vehicles [21], [22], [23].

**Figure 1:**
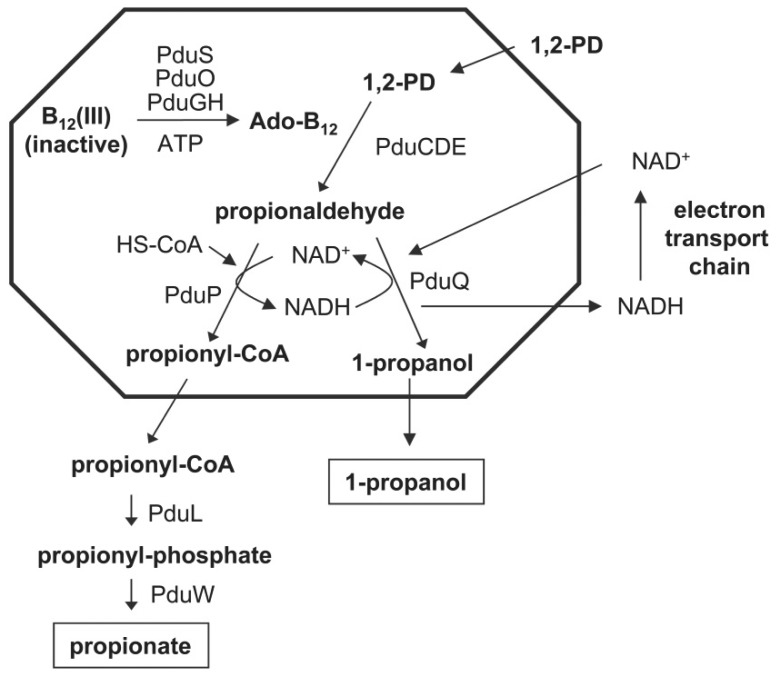
Model for the Pdu microcompartment and the role of PduQ. The Pdu MCP consists of a protein shell that encapsulates enzymes and cofactors for metabolizing 1,2-propanediol. The shell is thought to be made from 9 proteins (PduABB'JKMNTU). Encapsulated within are enzymes for the activation of B_12_(III) to coenzyme B_12_ (Ado-B_12_) (PduS-O-GH) as well as three 1,2-PD degradative enzymes: Ado-B_12_-dependent diol dehydratase (PduCDE), propionaldehyde dehydrogenase (PduP) and 1-propanol dehydrogenase (PduQ). The proposed function of the Pdu MCP is to sequester propionaldehyde and channel it to downstream enzymes in order to prevent toxicity and DNA damage. The PduQ enzyme recycles NADH to NAD^+^ internally within the MCP and this is needed for the activity of the PduP enzyme (this study). Alternatively, NADH can be recycled to NAD^+^ by the electron transport chain although at a slower rate compared to internal recycling by PduQ. This latter process is thought to require movement of NAD^+^/NADH through specific pores that span the protein shell.

*Salmonella enterica* produces a MCP for coenzyme B_12_-dependent 1,2-PD utilization (Pdu MCP) [12]. 1,2-PD is a major product of the anaerobic degradation of common plant sugars rhamnose and fucose and is thought to be an important carbon and energy source in anoxic environments [24]. The Pdu MCP consists of a protein shell that encapsulates enzymes and cofactors used for metabolizing 1,2-PD [25]. Twenty-four genes for 1,2-PD utilization (*pdu*) are found in a contiguous cluster (*pocR*, *pduF* and *pduABB'CDEGHJKLMNOPQSTUVWX*) [12], [26], [27]. The *pdu* locus encodes enzymes for 1,2-PD degradation (PduCDELPQW) [12], [25], [28], [29], [30], [31], the conversion of cobinamide and cobalamin to coenzyme B_12_ (PduOSX) [32], [33], [34], [35], [36], [37], the reactivation of diol dehydratase (PduGH) [38], [39], eight proteins that likely form the shell of Pdu MCP (PduABB'JKNTU) [12], [25], [40], [41], [42] and one structural protein (PduM) that might also be a shell component [43]. The pathway of 1,2-PD degradation begins with the conversion of 1,2-PD to propionaldehyde by coenzyme B_12_-dependent diol dehydratase (PduCDE) [26], [28] (Fig. 1). Propionaldehyde is then converted to propionate by coenzyme A (CoA)-dependent propionaldehyde dehydrogenase (PduP) [29], phosphotransacylase (PduL) [30] and propionate kinase (PduW) [31], or to 1-propanol probably by a putative propionaldehyde dehydrogenase (PduQ) [12]. The proposed function of the Pdu MCP is to sequester propionaldehyde produced by diol dehydratase (PduCDE) and channel it to propionaldehyde dehydrogenase (PduP) in order to minimize toxicity and DNA damage [6], [7], [12], [41]. Current models for the Pdu MCP propose that its protein shell selectively retains propionaldehyde while allowing the entrance of required enzyme substrates and cofactors as well as the egress of metabolic products [3], [44], [45]. Metabolite movement into and out of the Pdu MCP is proposed to occur through centrally located pores in the BMC-domain proteins (PduABB'JKTU) that comprise the bulk of its shell [44], [45], [46], [47]. Based on studies with several different systems, it has been shown that the pores in different BMC domain proteins vary in size and chemical properties suggesting that different shell proteins have pores that act as conduits for particular metabolites [44], [47], [48], [49], [50], [51].

Our prior studies showed that the PduP propionaldehyde dehydrogenase, which catalyzes the conversion of propionaldehyde+NAD^+^+HS-CoA→propionyl-CoA+NADH, is encapsulated within the Pdu MCP [29]. The substrate for PduP (propionaldehyde) is generated internally within the MCP, but how this enzyme is supplied with NAD^+^ and HS-CoA is unknown. In this study, we identify the PduQ enzyme is an iron-dependent alcohol dehydrogenase used for 1,2-PD degradation by *Salmonella*. We also show that PduQ is a new component of the Pdu MCP that plays an important role in regenerating NAD^+^ from NADH internally within the MCP, although results suggest other mechanisms such as specific pores may also be used. The findings presented here are the first to show the importance of internal recycling for cofactor homeostasis within a bacterial MCP.

## Materials and Methods

### Bacterial strains and growth conditions

The bacterial strains used in this study are listed in Table 1. The rich media used were lysogeny broth (LB) also known as Luria-Bertani/Lennox medium (Difco, Detroit, MI) [52] and Terrific Broth (TB) (MP Biomedicals, Solon, OH) [53]. The minimal medium used was no-carbon-E (NCE) medium [54].

**Table 1:** Bacterial strains used in this study.

Species and strain	Genotype	Source
*E. coli*		
BE237	BL21(DE3) RIL/pET-41a	Lab collection
BE272	BL21(DE3) RIL/pTA925-*pduP*	Lab collection
BE1421	BL21(DE3) RIL/pET-41a-His_6_-*pduP*	Lab collection
BE1422	BL21(DE3) RIL/pET-41a-*pduP*-His_6_	Lab collection
BE1500	BL21(DE3) RIL/pET-41a-*pduQ*	This work
BE1501	BL21(DE3) RIL/pET-41a-His_6_-*pduQ*	This work
BE1502	BL21(DE3) RIL/pET-41a-*pduQ*-His_6_	This work
*S. enterica* serovar Typhimurium LT2		
BE287	LT2/pLAC22	Lab collection
BE182	Δ*pduAB652*	Lab collection
BE192	Δ*pduQ660*	Lab collection
BE324	Δ*pduAB652*, Δ*pduQ660*	Lab collection
BE903	Δ*pduQ688*::frt	This work
BE919	Δ*pduQ688*::frt/pLAC22	This work
BE942	Δ*pduQ688*::frt/pLAC22-*pduQ*	This work
BE1651	Δ*pduQ688*::frt/pLAC22-*ZmadhB*	This work
BE1764	LT2/pLAC22-*ZmadhB*	This work

### Chemicals and reagents

Antibiotics, vitamin B_12_ (CN-B_12_, CN-Cbl), DNase I, NAD^+^, NADH, NADP^+^, and NADPH were from Sigma Chemical Company (St. Louis, MO). Isopropyl β-D-1-thiogalactopyranoside (IPTG) was from Diagnostic Chemicals Limited (Charlotteville PEI, Canada). *KOD* DNA polymerase was from Novagen (Cambridge, MA). *Taq* DNA polymerase, restriction enzymes, and T4 DNA ligase were from New England Biolabs (Beverly, MA). Bacterial protein extraction reagent (B-PER II) was from Pierce (Rockford, IL). Other chemicals were from Fisher Scientific (Pittsburgh, PA).

### Construction of plasmids and a *pduQ*-deletion mutant

The *pduQ* gene was amplified from the genomic DNA of *S. enterica* by PCR and then cloned into pET-41a vector (Novagen) using *Nde*I and *Hind*III restriction sites incorporated into the PCR primers as previously described [55]. The genes for the production of His_6_-PduQ and PduQ-His_6_ were similarly cloned with the sequence encoding the His_6_-tag incorporated into the PCR primers. The resulting plasmids pET-41a-*pduQ*, pET-41a-His_6_-*pduQ*, as well as the pET-41a-*pduQ*-His_6_ were introduced into *Escherichia coli* BL21 (DE3) RIL (Stratagene, La Jolla, CA). The native *pduQ* gene from *S. enterica* and the *adhB* gene from *Zymomonas mobilis* [56] were cloned into pLAC22 for complementation experiments. Vector pLAC22 allows tight regulation of protein production by IPTG [57]. All of the above inserts were verified by DNA sequencing.

The *pduQ* deletion mutant was constructed by a PCR-based method [58]. The deletion removed nearly the entire *pduQ* coding sequence, but left predicted translation signals of the adjacent genes (*pduP* and *pduS*) intact. The deletion of *pduQ* was verified by PCR as described previously [58] and by genomic DNA sequencing [40].

### P22 transduction

Transductional crosses were performed as described using P22 HT105/1 *int*-210, a mutant phage that has high transducing ability [59]. Transductants were tested for phage contamination and sensitivity by streaking on green plates against P22 H5.

### Growth of expression strains and purification of PduQ-His_6_

Four hundred ml of TB containing 25 µg/ml kanamycin in a 1 liter baffled Erlenmeyer flask was inoculated with 2 ml of an overnight TB culture of BE1502 and the cells were cultivated at 37°C with shaking at 275 rpm. When the culture reached an optical density at 600 nm of about 0.5, IPTG was added at 0.5 mM. The culture was incubated for additional 20 h at 16°C and 250 rpm shaking. Cells were then harvested by centrifugation at 4°C and 6,000×*g* for 10 min and used immediately for protein purification under anaerobic or aerobic conditions as previously described except that the buffer used was 50 mM potassium phosphate, pH 8.5, 300 mM NaCl, 5 mM β-mercaptoethanol, 0.4 mM AEBSF [4-(2-aminoethyl) benzenesulfonyl fluoride-HCl] [32]. Control strains BE237, BE1500 were grown in parallel with expression strain BE1502 and previously reported procedures were used for preparing whole-cell extracts and soluble fractions [32].

### SDS-PAGE and Western blots

Protein concentration was determined using Bio-Rad (Hercules, CA) protein assay reagent with bovine serum albumin (BSA) as a standard. SDS-PAGE was performed using Bio-Rad 10–20% gradient Tris-HCl ready gels. Protein bands were visualized by staining with Bio-Safe Coomassie Stain (Bio-Rad). For Western blots, the proteins on SDS-PAGE gels were transferred to polyvinylidene fluoride (PVDF) membranes and probed using SuperSignal West Pico Chemiluminescent Substrate (Pierce, Rockford, IL) according to the manufacturer's instructions using commercially prepared primary rabbit antisera (Genscript, Piscataway, NJ) against synthetic peptide ADNRISVFSEITPD (present at positions 51–64 in PduQ of *S. enterica*) at the final concentration of 0.5 µg/ml, and secondary goat anti-rabbit IgG-HRP (Santa Cruz Biotechnology, Santa Cruz, CA) at 40 ng/ml.

### Enzyme assays

Enzyme assays were performed under anaerobic conditions as described [32]. All reactions were initiated by addition of NADH or NAD^+^ to assay mixtures except where stated otherwise. Propionaldehyde reduction assays contained 100 mM Na_2_HPO_4_-NaH_2_PO_4_ (pH 7.0), 0.4 mM NADH, 150 mM propionaldehyde and the amount of enzyme indicated in the text. 1-propanol oxidation assays contained 100 mM Tris-HCl (pH 9.0), 2 mM NAD^+^, 800 mM 1-propanol and enzyme as indicated. The interconversion between propionaldehyde and 1-propanol was monitored spectrophotometrically by measuring the absorbance change at 340 nm due to consumption or formation of NADH and quantified using Δε_340_ = 6.22 mM^−1^ cm^−1^. PduP activities in anaerobically purified Pdu MCPs were assayed in 100 mM Na_2_HPO_4_-NaH_2_PO_4_ (pH 7.0), 200 mM 1,2-PD, 10 mM propionaldehyde, 40 µM NAD^+^, and 100 µM HS-CoA by monitoring the formation of thioester bonds at 232 nm with Δε_232_ = 4.5 mM^−1^ cm^−1^.

### Metal analysis

The content of Fe in the purified PduQ was determined by a high-resolution double-focusing inductively coupled plasma mass spectrometry (ICP-MS) using a Finnigan Element 1 (Thermo Scientific) operated at medium resolution (*m*/Δ*m* = 4,000) as described in a previous report [60]. Prior to metal analysis samples were applied to a Sephadex™ G-25 PD-10 desalting column (GE Healthcare, Piscataway, NJ) pre-equilibrated with MilliQ water (resistivity >18 mΩ) and eluted with MilliQ water inside an anaerobic chamber. Eluates were collected in metal-free tubes (Labcon, Petaluma, CA). As a control, water was used in place of purified PduQ and collected in the same volume range from a parallel PD-10 column. Ga was used as an internal standard to quantify the elemental concentrations of interest.

### Growth studies

Growth studies were performed using a Synergy HT Microplate reader (BioTek, Winooski, VT) as previously described [30]. For complementation studies, IPTG was used at 10 µM or as indicated in the text to induce expression of genes cloned into pLAC22. The growth curves were all repeated at least three times in triplicate and representative curves are shown. Doubling times were calculated from semilog plots as 0.693/(2.303×slope of the linear region of the plot).

### Electron microscopy, Pdu MCP purification and MALDI-TOF MS-MS

Electron microscopy was carried out as previously described [43]. Pdu MCPs were isolated as described except that the growth media additionally contained 50 µM ferric citrate [43]. The tandem mass spectroscopy was performed as previously described [32].

### His-tag protein affinity pull-down assays

The soluble cell lysates containing recombinant His-tagged baits were applied to the pre-equilibrated Ni-NTA column, and the column was washed with 50 mM potassium phosphate, pH 8.5, 300 mM NaCl, 1 mM DTT, 0.5 mM AEBSF and 100 mM imidazole. A soluble cell lysate containing a native recombinant protein prey was loaded onto the column which was again washed with the buffer described above. The His-tagged bait and any bound proteins were eluted with 50 mM potassium phosphate, pH 8.5, 300 mM NaCl, 1 mM DTT, 0.5 mM AEBSF and 300 mM imidazole and the eluate was analyzed by SDS-PAGE.

## Results

### Sequence analysis

Over 100 PduQ homologues present in GenBank are associated with 1,2-PD degradation based on gene proximity. According to amino acid sequence, the PduQ enzyme of *S. enterica* belongs to the family of iron-dependent group III alcohol dehydrogenases (Pfam0456) [61]. PduQ also possesses an NAD-binding motif (^89^GGG^91^) [62] and an iron-binding motif (^241^GX_2_HX_2_AHX_2_GX_5_PHG^259^ where X denotes any amino acid) [63] (Fig. S1). Although PduQ contains another classic NAD(P)-binding fingerprint (^13^GXGXX[A/G]^18^), previous studies indicated that this motif was not involved in binding NAD(P)^+^ in group III alcohol dehydrogenases [64].

### Expression and purification of PduQ-His_6_ protein

Based on its similarity to alcohol dehydrogenases, PduQ was previously proposed to be a 1-propanol dehydrogenase involved in 1,2-PD degradation by *Salmonella* [12]. However, the function of PduQ has not been investigated experimentally. To further characterize this enzyme, *E. coli* strain BE1502 was constructed to produce high levels of C-terminally His_6_-tagged PduQ protein via a T7 expression system. Strain BE1502 produced relatively large amounts of protein near the expected molecular mass of PduQ-His_6_ (40.3 kDa) (Fig. S2). PduQ-His_6_ was purified under both aerobic and anaerobic conditions by Ni-NTA affinity chromatography. Based on SDS-PAGE anaerobically purified PduQ-His_6_ protein was about 95% homogenous (Fig. S2, lane 4), and a similar level of purity was obtained for aerobically purified enzyme.

### In vitro catalytic activities of the PduQ-His_6_ enzyme

PduQ-His_6_ purified under strictly anaerobic conditions was tested for propionaldehyde reductase and 1-propanol dehydrogenase activities after each purification step (Table 2). The whole-cell extract from the PduQ-His_6_ expression strain (BE1502) exhibited about 24.2-fold higher propionaldehyde reductase activity (2.9±0.2 µmol min^−1^ mg^−1^) than did extracts from the control strain carrying the expression plasmid without insert (BE237) (0.12±0.03 µmol min^−1^ mg^−1^). Purification of the PduQ-His_6_ protein by anaerobic Ni-NTA chromatography increased the propionaldehyde reductase specific activity approximately 19.1-fold to 55.5±4.2 µmol min^−1^ mg^−1^. Assays showed that the PduQ-His_6_ protein also catalyzed the reverse reaction, the oxidation of 1-propanol to propionaldehyde. Whole cell extracts of the expression strain BE1502 had 0.7±0.1 µmol min^−1^ mg^−1^ 1-propanol dehydrogenase activity, and control extracts from BE237 had 0.42±0.08 µmol min^−1^ mg^−1^ activity. Purification of the PduQ-His_6_ protein by anaerobic Ni-NTA chromatography increased the 1-propanol dehydrogenase specific activity about 7.3-fold to 5.1±0.7 µmol min^−1^ mg^−1^. The differences in the activity increases of purified PduQ with different assay methods (propionaldehyde reductase and 1-propanol dehydrogenase) can be explained by differences in propionaldehyde reductase and 1-propanol dehydrogenase background activities present in crude cell extracts. Subtracting the background (0.1 and 0.4 µmol min^−1^ mg^−1^) (Table 2) the increases in specific activity with the different assay methods were 19.8- and 17-fold. Additional controls indicated that the C-terminal His_6_-tag had no major effect on PduQ catalytic activity; whole-cell extracts and soluble fractions of BE1502 and BE1500, which express PduQ-His_6_ and native recombinant PduQ, had similar enzymatic activities.

**Table 2:** Anaerobic purification of PduQ-His_6_.

Purification step	Total protein (mg)	Propionaldehyde reductase			1-propanol dehydrogenase		
		Specific activity (µmol min^−1^ mg^−1^)	Total activity (µmol min^−1^)	Purification fold	Specific activity (µmol min^−1^ mg^−1^)	Total activity (µmol min^−1^)	Purification fold
Whole-cell extract	403.6	2.9±0.2^a^	1170	1	0.7±0.1^a^	282.5	1
Soluble fraction	246.4	3.1±0.3^a^	763.8	1.1	0.8±0.2^a^	197.1	1.1
Ni-NTA eluate	4.4	55.5±4.2	244.2	19.1	5.1±0.7	22.4	7.3

a The enzyme activities measured in the control strain (plasmid without insert) were not subtracted from the values shown in the table.

### Aerobic purification substantially inactivates PduQ-His_6_

PduQ-His_6_ was also purified under aerobic conditions. Aerobically purified PduQ-His_6_ lost about 80% of both propionaldehyde reductase and 1-propanol dehydrogenase activities compared to the anaerobically purified enzyme (when assays were conducted immediately after purification). Further studies showed that PduQ lost measureable activity after several hours of air exposure (2 h for aerobically purified enzyme and 5 h for anaerobically purified PduQ). These results indicated that PduQ is oxygen sensitive.

### PduQ reaction requirements, linearity and pH optima

To determine the PduQ reaction requirements, key assay components were individually omitted. For propionaldehyde reduction, there was no detectable activity in the absence of NADH, propionaldehyde, or anaerobically purified PduQ-His_6_. In the 1-propanol dehydrogenase assays, no activity was measurable in the absence of NAD^+^, 1-propanol, or anaerobically purified PduQ-His_6_. The effects of PduQ-His_6_ concentration on its enzymatic activities were also determined. Propionaldehyde reductase was proportional to PduQ-His_6_ concentration from 10 to 95.5 nM when 0.4 mM NADH and 150 mM propionaldehyde were used as substrates. Linear regression yielded an *R* ^2^ value of 0.9981. 1-propanol dehydrogenase activity was linear from 0.16 to 1.4 µM PduQ-His_6_ with an *R* ^2^ value of 0.9994 when the assay mixture contains 2 mM NAD^+^ and 800 mM 1-propanol. Subsequent characterization of PduQ was done working within the linear ranges of these assays. The pH optima for PduQ-His_6_ were determined in 100 mM Na_2_HPO_4_-NaH_2_PO4 (pH 6.0–7.5), 100 mM Tris-HCl (pH 7.5–9.0) and 100 mM Glycine-NaOH (pH 9.0–10.0). The anaerobically purified PduQ-His_6_ exhibited maximal activity for propionaldehyde reduction at pH 7.0 while the maximal 1-propanol dehydrogenase activity was achieved at pH 9.0 (Fig. S3), which is consistent with the pH preference of several other alcohol dehydrogenases [61].

### NAD(H)/NADP(H) preference of PduQ-His_6_

To determine the cofactor specificity, anaerobically purified PduQ-His_6_ was assayed for both 1-propanol dehydrogenase and propionaldehyde reductase activities in the presence of either NADH or NADPH at 0.4 mM, or NAD^+^ or NADP^+^ at 2 mM. Results showed that anaerobically purified PduQ-His_6_ preferred NAD^+^/NADH as co-substrates. The relative activities with NADP^+^ and NADPH were 11% (1-propanol dehydrogenase) and 13% (propionaldehyde reductase), respectively.

### Characterization of Fe ion in PduQ-His_6_

ICP-MS was used to determine the iron content of PduQ-His_6_ (Fig. S4). The anaerobically purified PduQ-His_6_ protein contained 1.01±0.04 Fe-atom per monomeric unit while the aerobically purified PduQ-His_6_ protein contained 0.18±0.02 Fe-atom per monomeric unit, indicating that iron was lost in the presence of oxygen. In addition, the PduQ catalytic activities were almost totally inhibited by 1 mM EDTA. In conjunction with the finding that aerobically purified PduQ-His_6_ has about 20% relative activity compared to anaerobically purified enzyme (above), we infer that oxygen-labile iron is required for catalysis by PduQ.

### Kinetic analysis of PduQ-His_6_ activities

Steady-state kinetic studies for propionaldehyde reduction and 1-propanol oxidation were performed using anaerobically purified PduQ-His_6_ with varied concentrations of one substrate and a fixed excess concentration of the other substrate. Kinetic parameters were obtained by non-linear curve fitting to the Michaelis-Menten equation *v* = *V*_max_ [S]/(*K_m_*+[S]) using GraphPad Prism 5 Software (GraphPad Software, San Diego, CA). For propionaldehyde reduction, the *K_m_* values for NADH and propionaldehyde were 45.3±5.3 µM and 16.0±2.0 mM, respectively (Table 3). The enzyme *V*_max_ was 77.7±3.0 or 81.3±4.5 µmol min^−1^ mg^−1^ when propionaldehyde or NADH was held at 150 mM and 400 µM, respectively, and the other substrate was varied. For 1-propanol oxidation, non-linear regression indicated a *K_m_* of 267.5±22.3 µM and 95.8±9.2 mM for NAD^+^ and 1-propanol, respectively (Table 3). The enzyme *V*_max_ was 9.2±0.7 or 8.4±0.8 µmol min^−1^ mg^−1^ when the 1-propanol or NAD^+^ was held at fixed concentrations of 800 mM and 2 mM, respectively, and the other substrate was varied.

**Table 3:** Kinetic parameters for propionaldehyde reduction and 1-propanol oxidation by anaerobically purified PduQ-His_6_.

Reaction	Variable substrate	*K_m_* a (µM)	*V*_max_ a (µmol min^−1^ mg^−1^)	*k_cat_* (s^−1^)	*k_cat_/K_m_* (µM^−1^ s^−1^)
Propionaldehyde reduction^b^	NADH	45.3±5.3	77.7±3.0	52.2	1.15
	propionaldehyde	(16.0±2.0 )×10^3^	81.3±4.5	54.6	3.41×10^−3^
1-propanol oxidation^c^	NAD^+^	267.5±22.3	9.2±0.7	6.18	2.31×10^−2^
	1-propanol	(95.8±9.2 )×10^3^	8.4±0.8	5.64	5.89×10^−5^

a The values of *K_m_* and *V*_max_ were from non-linear regression using GraphPad Prism 5. The results shown are based on two independent experiments in which assays were done in triplicate.

### PduQ is a component of Pdu MCP

**Figure 2:**
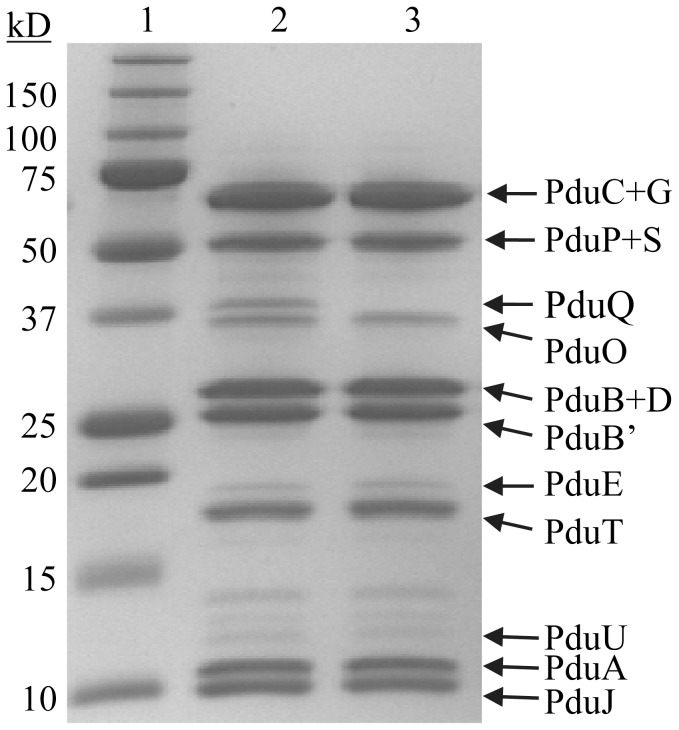
PduQ is a component of the Pdu MCP. Panel A: 10–20% SDS-PAGE gel stained with Bio-Safe Coomassie. Lane 1, molecular mass markers; lane 2, 10 µg Pdu MCPs purified from wild-type *Salmonella*; lane 3, 10 µg Pdu MCPs purified from a *pduQ* deletion mutant BE903. Panel B: Western blot with antisera against PduQ peptide (51–64). Lane 1, 10 µg Pdu MCPs purified from wild-type *Salmonella*; lane 2, 10 µg Pdu MCPs purified from *ΔpduQ* mutant BE903.

To determine the cellular location of PduQ, MCPs purified from wild-type *S. enterica* and *pduQ* deletion mutant BE903, were analyzed by SDS-PAGE, Western blot, MALDI-TOF MS-MS and enzymatic assays. A band near the expected molecular mass of PduQ (39.5 kDa) was observed on an SDS-PAGE gel of MCPs purified from the wild-type strain (Fig. 2A lane 2) but was absent in MCPs purified from the *pduQ* deletion mutant (Fig. 2A lane 3). Digestion of this band with trypsin followed by MALDI-TOF MS-MS identified three sequences: MNTFSLQTR·L (1113.56), R·FNAGVPR·A (760.41), R·IPAMVQAALADVTLR·T (1584.86), and all of them matched the PduQ protein. The Mowse Score was 96 indicating a high likelihood of a correct identification. In addition, Western blots with antisera against PduQ peptide (51–64) detected a band near 39 kDa in wild-type MCPs (Fig. 2B lane 1), while no band was detected from the Δ*pduQ* MCPs (Fig. 2B lane 2). Moreover, the NADH-dependent reduction of propionaldehyde occurred at rates of 2.48±0.19 or 0.24±0.03 µmol min^−1^ mg^−1^ in anaerobically purified wild-type MCPs and crude cell extracts, respectively, showing the PduQ was enriched in the purified MCPs. As expected no activity was detected in *ΔpduQ* MCPs. We also found that the activity of PduQ in the purified MCP correlated well with the activity of the purified enzyme. The activity of PduQ in the purified MCP was 2.48±0.19 µmol min^−1^ mg^−1^. The specific activity of the purified enzyme was 55.5±4.2. This implies that PduQ is 4.5% of the total MCP protein. Our prior studies indicated that the band just below PduQ (PduO) is 3.6% of the total MCP protein [25] and PduQ band has similar intensity to PduO in the purified Pdu MCP on SDS-PAGE (Fig. 2A lane 2). PduP was previously estimated to make up about 8% of the total MCP protein [25]. This suggests an approximate stoichiometry of 2 PduP/PduQ. The *V*max values for PduP and PduQ were previously determined to be 85 and 77 U in the physiologically relevant directions. These values correlate reasonably well with each other and the estimated stoichiometry in light of potential variation of these activities in vivo compared to in vitro. Overall, the above results show that the PduQ protein is a component of Pdu MCPs.

### A *pduQ* deletion mutant forms normal appearing MCPs

Given that PduQ was found to be a component of the Pdu MCP, we tested the effect of a *pduQ* deletion mutation on MCP structure. Electron microcopy showed that about 85% of cells carrying a *pduQ* deletion (BE903) formed normal appearing MCPs. A representative image is shown in Fig. S5. We also used electron microscopy to compare MCPs purified from wild-type *Salmonella* and a *pduQ* deletion mutant and found them to be indistinguishable (Fig S6). We infer that PduQ does not play a major structural role in MCP formation. Prior studies showed that individually deleting the genes that encode four other MCP enzymes (the PduCDE diol dehydratase, the PduP propionaldehyde dehydrogenase, the PduO adenosyltransferase and the PduS cobalamin reductase) also had little effect on MCP structure so the findings with the *pduQ* mutant were not unusual [29], [32] (and unpublished).

### PduQ is required for maximal growth on 1,2-propanediol

**Figure 3:**
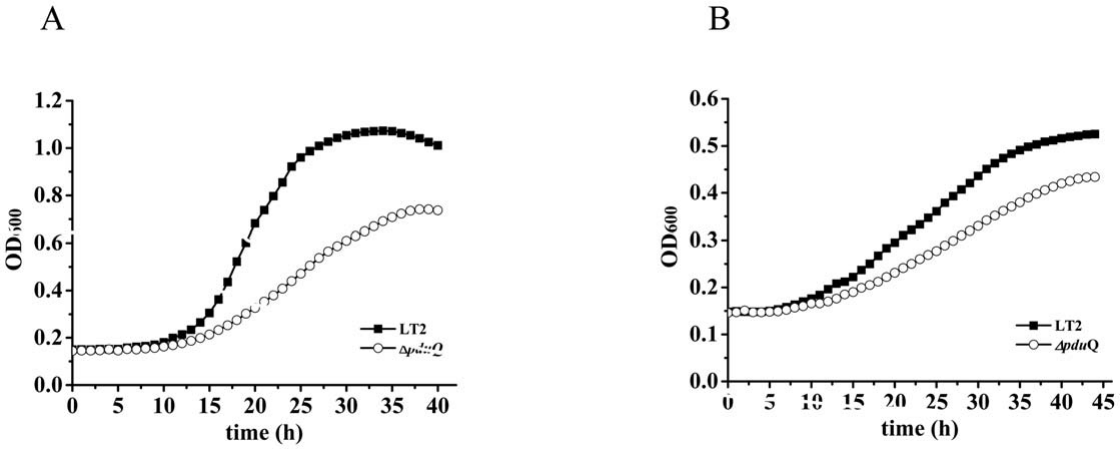
A *pduQ* deletion mutant grows slowly on 1,2-PD. Cells were grown on minimal 1,2-PD medium with saturating (150 nM) or limiting (20 nM) CN-Cbl (vitamin B_12_): panels A or B, respectively. Symbols meanings are indicated on the figure.

To investigate the function of PduQ *in vivo*, we compared the growth of a *pduQ* deletion mutant (BE903) to wild-type *Salmonella*. On 1,2-PD minimal medium supplemented with saturating CN-B_12_ (150 nM), the *pduQ* deletion mutant grew at 54% of the wild-type rate (doubling time of 4.4±0.16 h versus 8.2±0.26 h) and to a lower cell density (OD_600_ of 0.72 versus 1.04) (Fig. 3A). During growth on 1,2-PD with limiting CN-B_12_ (20 nM) the *ΔpduQ* mutant also grew at a slower rate than wild-type (79%) (doubling time of 14.4±0.66 h versus 18.3±0.89 h) and to lower cell density (OD_600_ of 0.43 versus 0.53) (Fig. 3B). The growth curves shown are representative of ≥3 experiments done in triplicate. Thus, results indicate that the PduQ enzyme is used for 1,2-PD degradation by *Salmonella*. Based on prior studies of 1,2-PD degradation and numerous other bacterial catabolic process, the expected role of PduQ would be to provide extra capacity (above that of the electron transport chain) for the regeneration of NAD^+^ from NADH. However, further studies described below indicate that the primary role of PduQ is to regenerate NAD^+^ from NADH internally within the Pdu MCP (Fig. 1).

### The growth defect of a *ΔpduQ* mutant can be complemented by PduQ but not by a homologous non-MCP associated Adh enzyme

**Figure 4:**
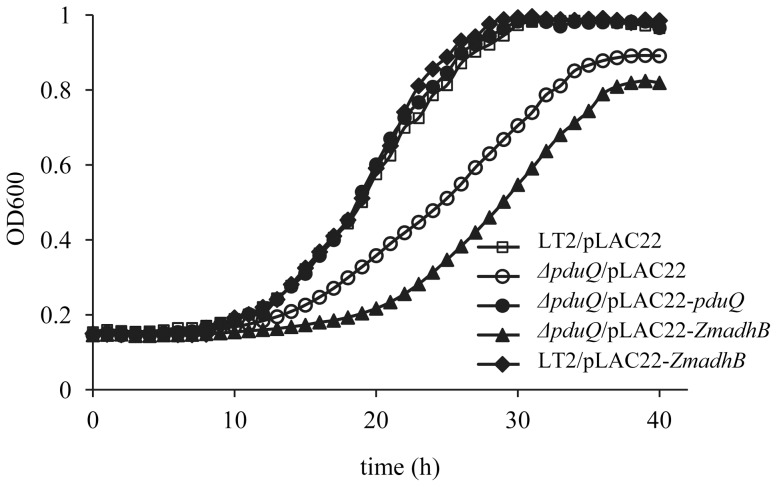
The Adh2 enzyme from *Z. mobilis* failed to complement a *pduQ* deletion mutant. Cells were grown in 1,2-PD minimal medium with 75 nM CN-Cbl (vitamin B_12_). 10 µM IPTG was used for induction of *pduQ* and 10–500 µm IPTG was used for induction for *ZmadhB* from pLAC22.

**Figure 5:**
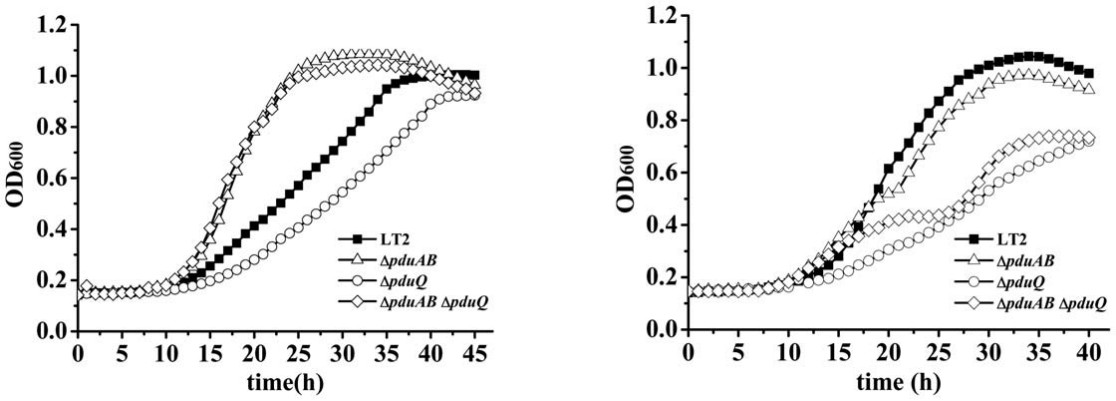
A *ΔpduQ* mutant is unimpaired for growth in a strain unable to form MCPs (*ΔpduAB*). Cells were grown on 1,2-PD minimal medium supplemented with 40 or 150 nM CN-Cbl (vitamin B_12_).

Complementation tests showed that production of PduQ from pLAC22 fully corrected the growth defect of the *ΔpduQ* mutant at CN-B_12_ levels ranging from 20–150 nM. This confirmed that the observed growth defect was due to the *pduQ* deletion, but not due to polarity or an unknown mutation. In contrast, the non-MCP associated Adh2 of *Z. mobilis* (*Zm*Adh2) was unable to correct the growth defect of a *pduQ* deletion mutant even though its enzymatic activity was similar (Fig. 4). In the growth curves shown, 10 µM IPTG was used for induction of both PduQ and *Zm*Adh2 which resulted in 0.52±0.04 and 0.63±0.03 µmol min^−1^ mg^−1^ propionaldehyde reductase activity, respectively. Furthermore, *Zm*Adh2 production was unable to correct the growth phenotype of a *ΔpduQ* deletion mutant when induced with IPTG at concentrations up to 500 µM. Further controls showed that expression of *Zm*Adh2 from pLAC22 had little effect on the growth of LT2 on 1,2-PD or was slightly stimulatory. The finding that *Zm*Adh2 was unable to correct the growth rate defect of a *pduQ* deletion mutant is contrary to the idea that the role of PduQ is simply to provide additional capacity for NADH oxidation. Moreover, this finding suggests that PduQ has a MCP specific function since the salient difference between PduQ and *Zm*Adh2 is that PduQ localizes to the MCP while *Zm*Adh2 is cytoplasmic. For the growth curves shown in Fig. 5 cultures were grown with 75 nM CN-B_12_ with 3 or more repetitions in triplicate. At 75 nM CN-B_12_ level the *ΔpduQ* mutant had a clear growth defect (this phenotype is reduced at lower CN-B_12_ concentrations) and the possibility of propionaldehyde toxicity is minimized [6].

### Preventing MCP formation genetically is epistatic to the growth defect of a *pduQ* mutation

We tested the effects of a *pduQ* deletion on growth of *Salmonella* on 1,2-PD in a background containing a *pduAB* deletion which was previously shown to prevent formation of the Pdu MCP [6], [41]. At lower levels of CN-B_12_, the *ΔpduQ* mutant grew slower than wild-type *Salmonella* and the *ΔpduAB* mutant grew faster as was previously reported [6], [41] (Fig. 5A). More importantly, the *ΔpduAB* strain and *ΔpduAB ΔpduQ* strain grew similarly. This indicates that growth defect of the *pduQ* deletion mutant depends on an intact MCP suggesting that the PduQ enzyme has a specific role in optimizing the function of the Pdu MCP. Furthermore, the finding that the *ΔpduAB ΔpduQ* mutant grew faster than wild-type indicates that the electron transport chain has sufficient capacity to support wild-type rates of 1,2-PD degradation in the absence of PduQ under the conditions used.

We note that the tests shown in Fig. 5A were done at 40 nM CN-B_12_. At this cofactor concentration, *ΔpduQ* has a clear phenotype and the chances of propionaldehyde toxicity (which is more severe in the *ΔpduAB* background [6], [41]) are minimized. Nonetheless, we also compared the growth of the *ΔpduAB* and the *ΔpduAB ΔpduQ* mutants on 1,2-PD minimal medium supplemented with saturating concentrations of CN-B_12_ (150 nM). At higher levels of CN-B_12_ the epistatic effect of *ΔpduAB* on *ΔpduQ* was partially obscured by a period of growth arrest (from about hour 17 to hour 27) resulting from accumulation of propionaldehyde to toxic levels as was previously reported [6], [41] (Fig. 5B). Even so, it can be seen that the growth of the *ΔpduAB ΔpduQ* strain was similar to that of the *ΔpduAB* strain until the onset of propionaldehyde toxicity.

### The PduQ enzyme can recycle NAD^+^ for PduP in purified MCPs

**Figure 6:**
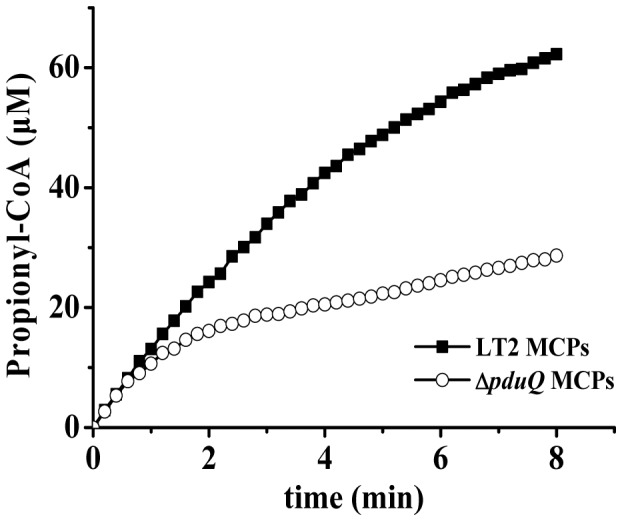
PduQ regenerates NAD^+^ for the PduP reaction in purified Pdu MCPs. The PduP activity of purified MCPs was measured by monitoring the absorbance increase at 232 nm due to propionyl-CoA formation. Limiting NAD^+^ (40 µM) was added in the assays. The propionyl-CoA produced by wild-type MCPs and *ΔpduQ* MCPs, respectively, reached 62.3 and 27.6 µM in 8 min. Because the concentration of propionyl-CoA produced by the wild-type exceeded the amount of NAD^+^ added to the reaction (the PduP reaction produces 1 NADH per propionyl-CoA formed) recycling of NADH to NAD^+^ is indicated.

To determine whether PduQ can recycle NAD^+^ for the PduP reaction in vitro, we measured the PduP activity in anaerobically purified MCPs from wild-type *Salmonella* and a *pduQ* deletion mutant. The method used to purify the Pdu MCPs was previously shown to yield intact MCPs [43]. Electron microscopy of both whole cells and purified MCPs indicated that *pduQ* deletion mutant had no major effect on MCP morphology (Fig. S5 and S6). In addition, the yield of MCPs and the protein ratios were similar for both wild-type and *Δpdu* MCP indicating that MCPs lacking PduQ were intact and stable. The PduP assay used measured the formation of propionyl-CoA by monitoring absorbance at 232 nm. Results showed that the concentration of propionyl-CoA produced by wild-type MCP reached 62.3 µM in 8 min which was higher than the concentration of NAD^+^ added to the assay (40 µM) (Fig. 6). Since the PduP reaction requires 1 molecule of NAD^+^ per molecule of propionyl-CoA formed, recycling of NADH to NAD^+^ by PduQ is indicated. In contrast, the concentration of propionyl-CoA produced by MCPs purified from a *pduQ* deletion mutant was 27.6 µM in the same time period (Fig. 6) which is substantially less than the amount of NAD^+^ added to the assay (40 µM). Results also showed the initial PduP reaction rates were similar in wild-type and *ΔpduQ* MCPs indicating both contained similar amounts of PduP. In addition, controls showed that there was no measureable PduP activity without added HS-CoA, NAD^+^ or propionaldehyde. Thus, these results show that purified MCPs recycle NAD^+^/NADH such that PduP converts NAD^+^ to NADH and PduQ converts NADH back to NAD^+^ which is consistent with internal recycling by PduQ.

### Binding interactions between PduQ and PduP

**Figure 7:**
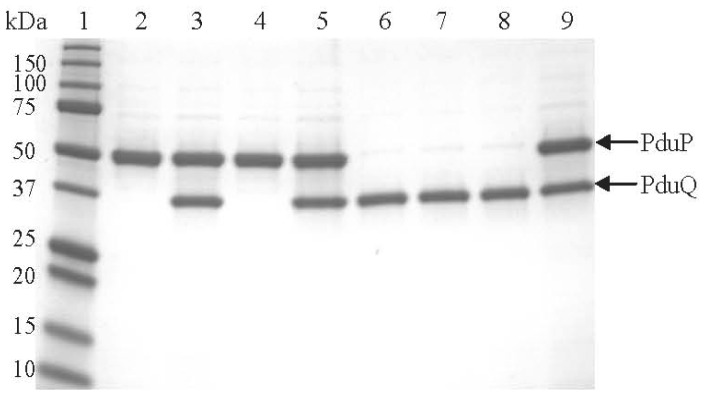
His-tag protein affinity pull-down assays indicated binding between PduQ and PduP. Lane 1, protein standards; lane 2, His_6_-PduP; lane 3, His_6_-PduP+PduQ; lane 4, PduP-His_6_; lane 5, PduP-His_6_+PduQ; lane 6, His_6_-PduQ; lane 7, His_6_-PduQ+PduP; lane 8, PduQ-His_6_; lane 9, PduQ-His_6_+PduP.

To examine the potential binding interactions between PduQ and PduP *in vitro*, His-tag pull-down assays were performed. Crude cell extracts containing His-tagged bait or potential target proteins were sequentially passed over a Ni-NTA affinity column and then the column was washed and eluted with buffers containing low and high imidazole concentrations, respectively. In this test, binding is indicated for proteins that co-elute with the His-tagged bait at high imidazole concentrations while proteins that do not bind the His-tagged bait pass through the column at low imidazole concentrations. SDS-PAGE demonstrated that native recombinant PduQ co-eluted with N- or C-terminally His-tagged PduP at high imidazole concentrations, and in a reciprocal experiment native recombinant PduP co-eluted with C-terminally His-tagged PduQ (Fig. 7). Control experiments showed that native recombinant PduQ and native recombinant PduP did not bind to the Ni-NTA column. Thus, these results indicate that PduQ and PduP bind to one another under the conditions used. The binding of PduQ and PduP is consistent with roles in regenerating NADH/NAD^+^ for one another (Fig. 1). In addition, prior studies localized PduP to the interior of the Pdu MCP [29]; hence, these results suggest PduQ is also a MCP lumen enzyme. We also note that native recombinant PduP did not co-elute with PduQ having a His-tag on its N-terminus (Fig. 7 lane 7). This suggests that the N-terminus of PduQ may be involved in PduQ-PduP binding and that the N-terminal His-tag interfered with this interaction.

## Discussion

Prior studies indicated that a 1-propanol dehydrogenase was involved in 1,2-PD degradation and that the role of this enzyme was to regenerate NAD(P)^+^ from NAD(P)H [12], [24]. Previous work also showed that a gene in the *pdu* operon (*pduQ*) encoded an enzyme with homology to known Adh enzymes [12]. The results presented in this report establish experimentally that the PduQ enzyme of *S. enterica* is an iron-dependent Adh used for 1,2-PD degradation. Purified PduQ catalyzed the conversion of propionaldehyde to 1-propanol and the reverse reaction with specific activities of 55.5±4.2 and 5.1±0.7 µmol min^−1^ mg^−1^, respectively. These rates are similar to those measured for other catabolic Adh enzymes [61]. We also showed that a *pduQ* deletion mutant was impaired for growth on 1,2-PD which provided in vivo evidence for its role in 1,2-PD degradation. In addition, in vitro studies showed that PduQ prefers NADH to NADPH as a co-substrate which is consistent with its role in regenerating NAD^+^ for the second step of 1,2-PD degradation (catalyzed by the PduP enzyme) which preferentially uses NAD^+^ [29]. Thus, results presented here in conjunction with previous work indicate that PduQ is a 1-propanol dehydrogenase used to regenerate NAD^+^ from NADH during 1,2-PD metabolism.

Our prior work showed that *S. enterica* uses a MCP for B_12_-dependent 1,2-PD degradation [12]. Subsequent proteomics studies identified many of the protein components of the Pdu MCP, but missed PduQ [25]. In this study, enzyme assays, SDS-PAGE, Western blot (Fig. 2) and MS-MS demonstrated that PduQ is a component of purified Pdu MCPs. The enzymatic activity of PduQ was enriched 10-fold in purified MCPs compared to crude cell extracts which is similar to results seen for two other MCP enzymes, PduCDE and PduP [25], [29]. The finding that PduQ co-purified with the Pdu MCP through a treatment involving detergent extraction and differential centrifugation indicates a tight association between PduQ and the Pdu MCP. In addition, His-tag pull-downs indicated that PduQ binds PduP which is known to localize to the lumen of the Pdu MCP. This suggests that PduQ is also a lumen enzyme. This idea is further supported by the fact that PduQ has a putative C-terminal targeting sequence of the type recently described for CcmN [11] and by the fact that luminal localization of enzymes is a conserved feature of bacterial MCPs [5], [45]. We point out that although the studies reported here favor luminal localization of PduQ they do not rule a tight association of PduQ with outer surface of the MCP shell. However, the internal localization of PduQ is not a requirement for PduQ to function in internal NAD^+^ recycling since even if PduQ is a shell component, NAD^+^ could be released into the MCP interior directly or through pores shown to cross the shell in crystal structures of shell proteins [45]. Importantly, the finding that PduQ is an MCP component has significant bearing on how PduQ functions in 1,2-PD degradation as further described below.

Studies presented here and previously indicate that the role of the PduQ enzyme in 1,2-PD degradation is to regenerate NAD^+^ from NADH for the PduP enzyme [24], [29] (Fig. 1). Thus, a limiting supply of NAD^+^ likely accounts for slow growth of the *pduQ* mutant on 1,2-PD. Based on analogy with other bacterial catabolic processes such as glycolysis, it would make sense to infer that the electron transport chain is unable to regenerate NAD^+^ at a rate sufficient to support maximal growth and the PduQ enzyme is needed to provide extra NADH-oxidation capacity. However, the association of PduQ with the Pdu MCP raises the question of whether PduQ produces NAD^+^ by oxidation of the general NADH pool (total NADH inside and outside the MCP) or whether it has a special role in oxidizing NADH to NAD^+^ internally within the MCP. The slow growth phenotype of the *pduQ* deletion mutant indicated that NAD^+^ recycling becomes limiting for growth in the absence of PduQ. Importantly, genetically disrupting the MCP shell restored the growth of a *pduQ* mutant to wild-type levels. This essentially eliminates the idea that PduQ works on the total NADH pool. If that were the case, breaking the MCP should not correct the growth defect of a *pduQ* mutant since it would still have a reduced capacity to recycle the general NADH pool. At the same time, this result supports internal recycling since the finding that breaking the shell corrects the growth defect of a *pduQ* mutant is what would be expected for internal NAD^+^ recycling by PduQ: breaking the shell of the Pdu MCP would allow free diffusion of NADH facilitating recycling to NAD^+^ via the electron transport chain and eliminating the need for PduQ which plays the primary role in oxidizing NADH internally within the MCP. In addition, results showed that the growth defect of a *ΔpduQ* mutant could be fully corrected by production of PduQ from a plasmid but not by production of the non-MCP associated iron-dependent Adh2 from *Z. mobilis* even though Adh2 has similar catalytic properties and was produced with similar specific activity [56] (Fig. 4). This argues against the idea the PduQ simply provides extra NADH oxidation capacity and supports the inference that PduQ has a MCP-specific function. Moreover, we also conducted in vitro studies with purified MCPs that showed PduQ can convert NADH to NAD^+^ to support the PduP reaction which is consistent with a role in internal NAD^+^ regeneration (Fig. 6). Thus, cumulatively, the in vivo and in vitro studies described above indicate that the PduQ enzyme functions to regenerate NAD^+^ from NADH internally within the Pdu MCP.

The finding that PduQ functions to recycle NAD^+^ internally within the Pdu MCP also bears on a key mechanistic question of MCP function. The shells of bacterial MCPs are proposed to act as diffusion barriers that help retain toxic or volatile metabolic intermediates and channel them to downstream enzymes (propionaldehyde in the case of the Pdu MCP). A critical question about MCP function is how substrates and cofactors are provided to the enzymes enclosed within their protein shells while toxic/volatile metabolic intermediates are retained. The studies reported here indicate that NAD^+^/NADH can be encapsulated within the Pdu MCP during assembly then enzymatically recycled internally (Fig. 1). A prior proposal which is based on fairly extensive crystallographic studies, suggests that specific pores in the shell mediate the transport of enzymatic cofactors into and out of bacterial MCPs [45], [50]. In this scenario, NAD^+^ is converted to NADH by the PduP enzyme. NADH exits the MCP through specific pores that span the shell and is oxidized to NAD^+^ in the cytoplasm by the electron transport chain. Lastly, NAD^+^ re-enters the MCP again through specific pores. Internal recycling could conceivably obviate the need for specific pores that mediate cofactor movement across the MCP shell [45], [50]. However, several lines of evidence suggest that pores are also important. In this report, we showed that a *pduQ* deletion mutant still grew at about 54% of the wild-type rate on 1,2-PD at saturating B_12_ (Fig. 4). This demonstrates that NADH can be converted to NAD^+^ at a substantial rate independently of PduQ during growth of *Salmonella* on 1,2-PD. It also suggests that NADH/NAD^+^ can move into and out of the MCP fairly efficiently since in the absence of PduQ the oxidation of NADH to NAD^+^ is expected to occur solely via the electron transport chain. This work and prior studies also found that the PduP enzyme is active in purified MCPs and its activity requires addition of NAD^+^ to assay mixtures showing that purified MCPs are permeable to NAD^+^ due to pores that traverse the shell [29]. In addition, crystallography has shown that the pores in MCP shell proteins vary in size and chemical properties suggesting substrate specificity and that some shell proteins have two crystal forms (pore closed and pore open) suggesting a gating mechanism [45], [48]. On an evolutionary basis, these properties are unlikely to be coincidental [50]. Thus, based on results reported here and previously, we propose that NADH/NAD^+^ homeostasis within the Pdu MCP is mediated by both internal recycling and specific pores. Importantly, this model could apply to a variety of enzymatic cofactors required by diverse MCPs

## Supporting Information

Figure S1 Sequence alignment by Clustal X2. EcFucO, 1,2-propanediol dehydrogenase from *Escherichia coli* (GI 16130706); KpDhaT, 1,3-propanediol dehydrogenase from *Klebsiella pneumoniae* (GI 940440); SeEutG, ethanol dehydrogenase from *Salmonella enterica* (GI 687647); ZmAdh2, Adh2 from *Zymomonas mobilis* (GI 56552492); SePduQ, 1-propanol dehydrogenase from *S. enterica* (GI 5069460). Boxed: Glycine-rich motif involved in NAD^+^-binding. Arrows: histidine residues that coordinate iron. Triangle: histidine residue essential for catalysis and thought to interact with the substrate.
(TIF)

Figure S2 SDS-PAGE analysis of the anaerobic purification of PduQ-His_6_ from *E. coli* BE1052. Lane 1, protein standards; lane 2, 10 µg whole-cell extract; lane 3, 10 µg soluble fraction; lane 4, 2 µg PduQ-His_6_ following Ni affinity chromatography. The gel was a Bio-Rad 10–20% gradient ready gel stained with Coomassie.
(TIF)

Figure S3 pH dependence of propionaldehyde reduction and 1-propanol oxidation activities catalyzed by PduQ-His_6_ of *S. enterica*. The maximal activities for propionaldehyde reduction and 1-propanol oxidation were achieved at pH 7.0 and 9.0, respectively. The buffers used were 100 mM Na_2_HPO_4_-NaH_2_PO_4_ at pH 6.0–7.5; 100 mM Tris-HCl at pH 7.5–9.0 and 100 mM Glycine-NaOH at pH 9.0–10.0.
(TIF)

Figure S4 Identification of Fe in purified PduQ-His_6_ using ICP-MS (cps: counts per second).
(TIF)

Figure S5 Electron microscopy of wild-type *Salmonella enterica* and a *pduQ* deletion mutant. Triangles point to microcompartments. A number of sections were examined and the cells shown are representative.
(TIF)

Figure S6 Electron microscopy of MCPs purified from wild-type *Salmonella enterica* and a *pduQ* deletion mutant. The image shown is a representative negative stain.
(TIF)
