# Supplementary figures and images for: The PduQ Enzyme Is an Alcohol Dehydrogenase Used to Recycle NAD+ Internally within the Pdu Microcompartment of Salmonella enterica
Source: PLoS One. 2012 Oct 15;7(10):e47144. doi: 10.1371/journal.pone.0047144 (PMC3471927; doi:10.1371/journal.pone.0047144)

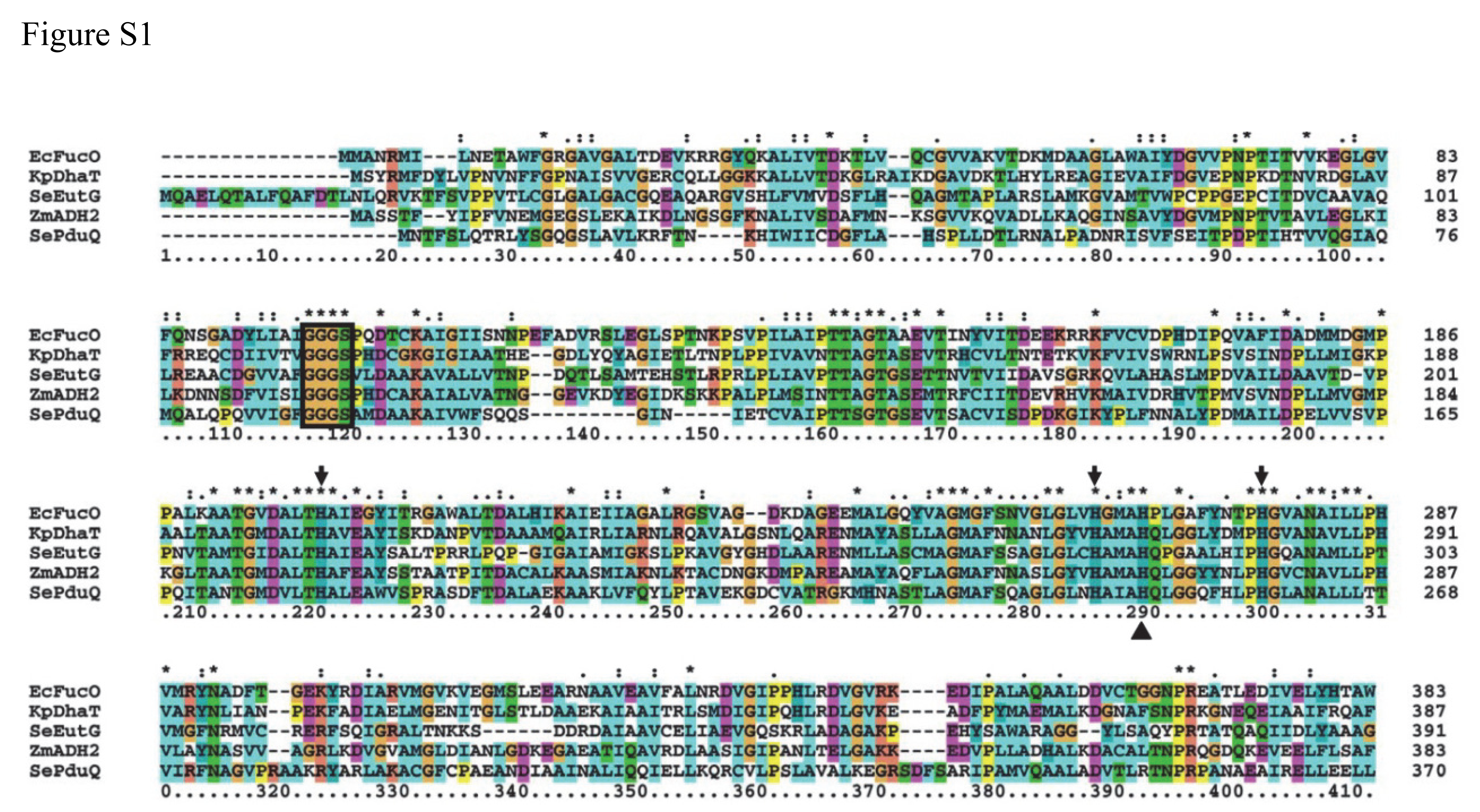

Supplement: Figure S1 — Sequence alignment by Clustal X2. EcFucO, 1,2-propanediol dehydrogenase from Escherichia coli (GI 16130706); KpDhaT, 1,3-propanediol dehydrogenase from Klebsiella pneumoniae (GI 940440); SeEutG, ethanol dehydrogenase from Salmonella enterica (GI 687647); ZmAdh2, Adh2 from Zymomonas mobilis (GI 56552492); SePduQ, 1-propanol dehydrogenase from S. enterica (GI 5069460). Boxed: Glycine-rich motif involved in NAD+-binding. Arrows: histidine residues that coordinate iron. Triangle: histidine residue essential for catalysis and thought to interact with the substrate. (TIF) [file pone.0047144.s001.tif]

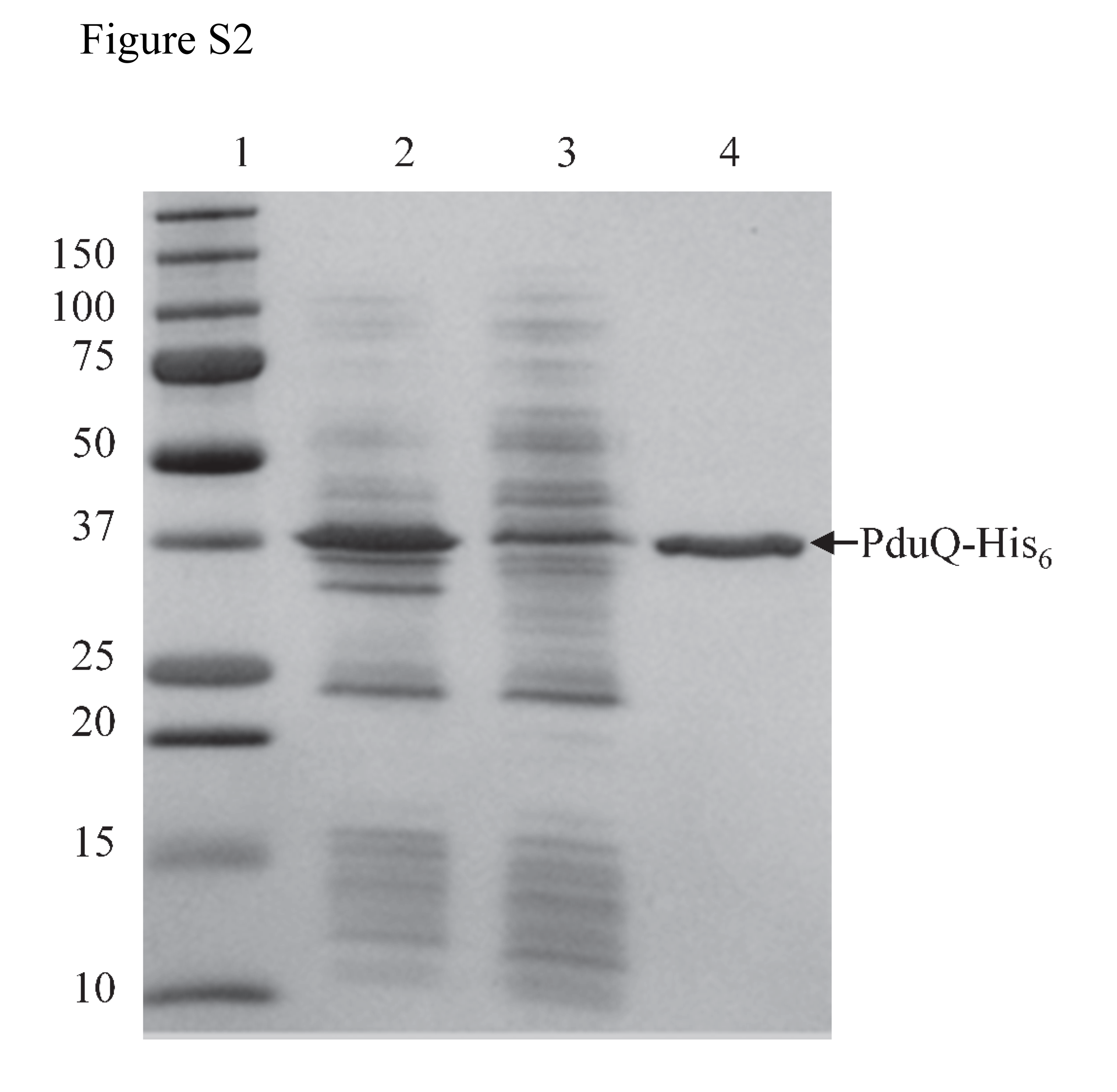

Supplement: Figure S2 — SDS-PAGE analysis of the anaerobic purification of PduQ-His6 from E. coli BE1052. Lane 1, protein standards; lane 2, 10 µg whole-cell extract; lane 3, 10 µg soluble fraction; lane 4, 2 µg PduQ-His6 following Ni affinity chromatography. The gel was a Bio-Rad 10–20% gradient ready gel stained with Coomassie. (TIF) [file pone.0047144.s002.tif]

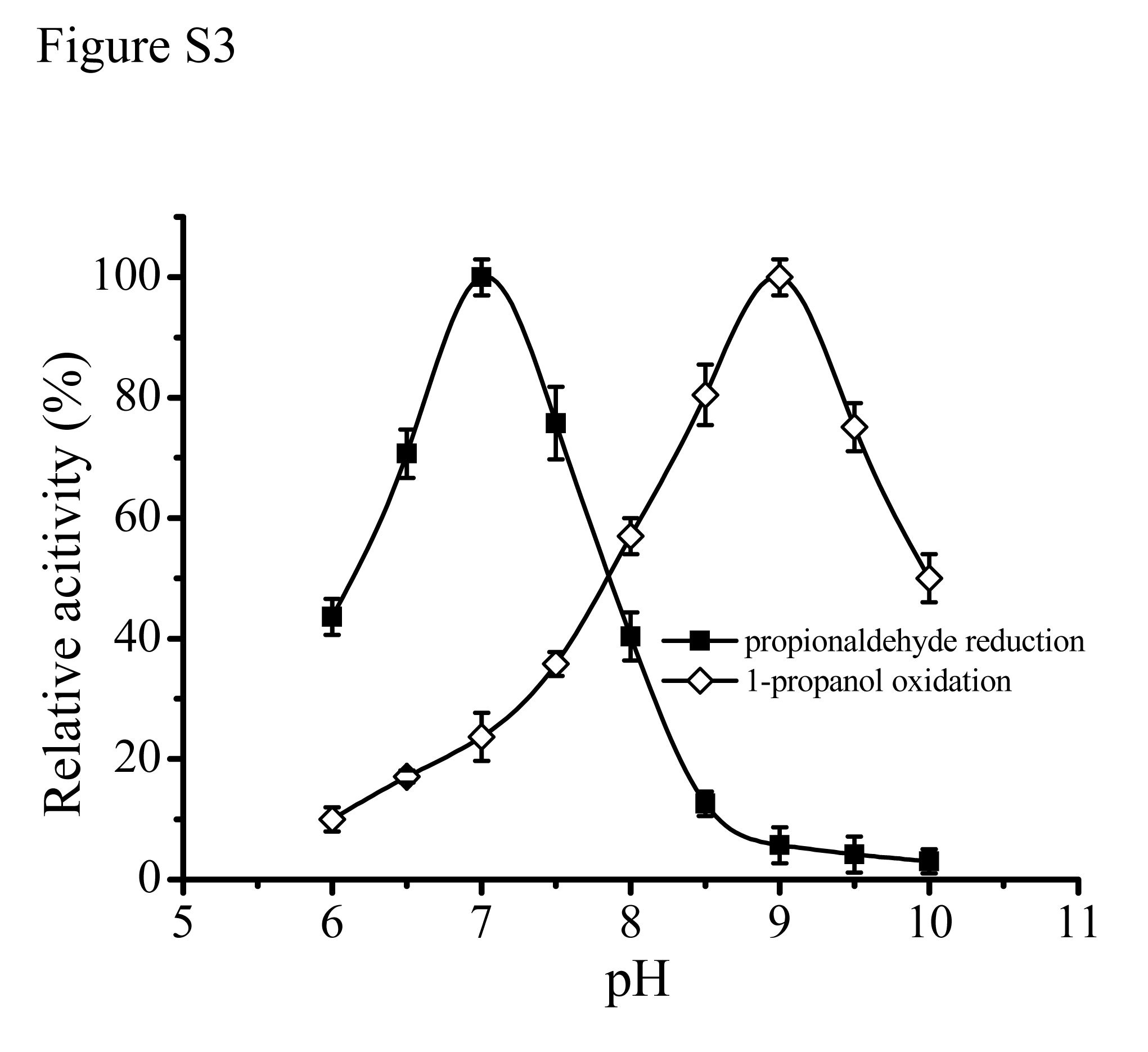

Supplement: Figure S3 — pH dependence of propionaldehyde reduction and 1-propanol oxidation activities catalyzed by PduQ-His6 of S. enterica. The maximal activities for propionaldehyde reduction and 1-propanol oxidation were achieved at pH 7.0 and 9.0, respectively. The buffers used were 100 mM Na2HPO4-NaH2PO4 at pH 6.0–7.5; 100 mM Tris-HCl at pH 7.5–9.0 and 100 mM Glycine-NaOH at pH 9.0–10.0. (TIF) [file pone.0047144.s003.tif]

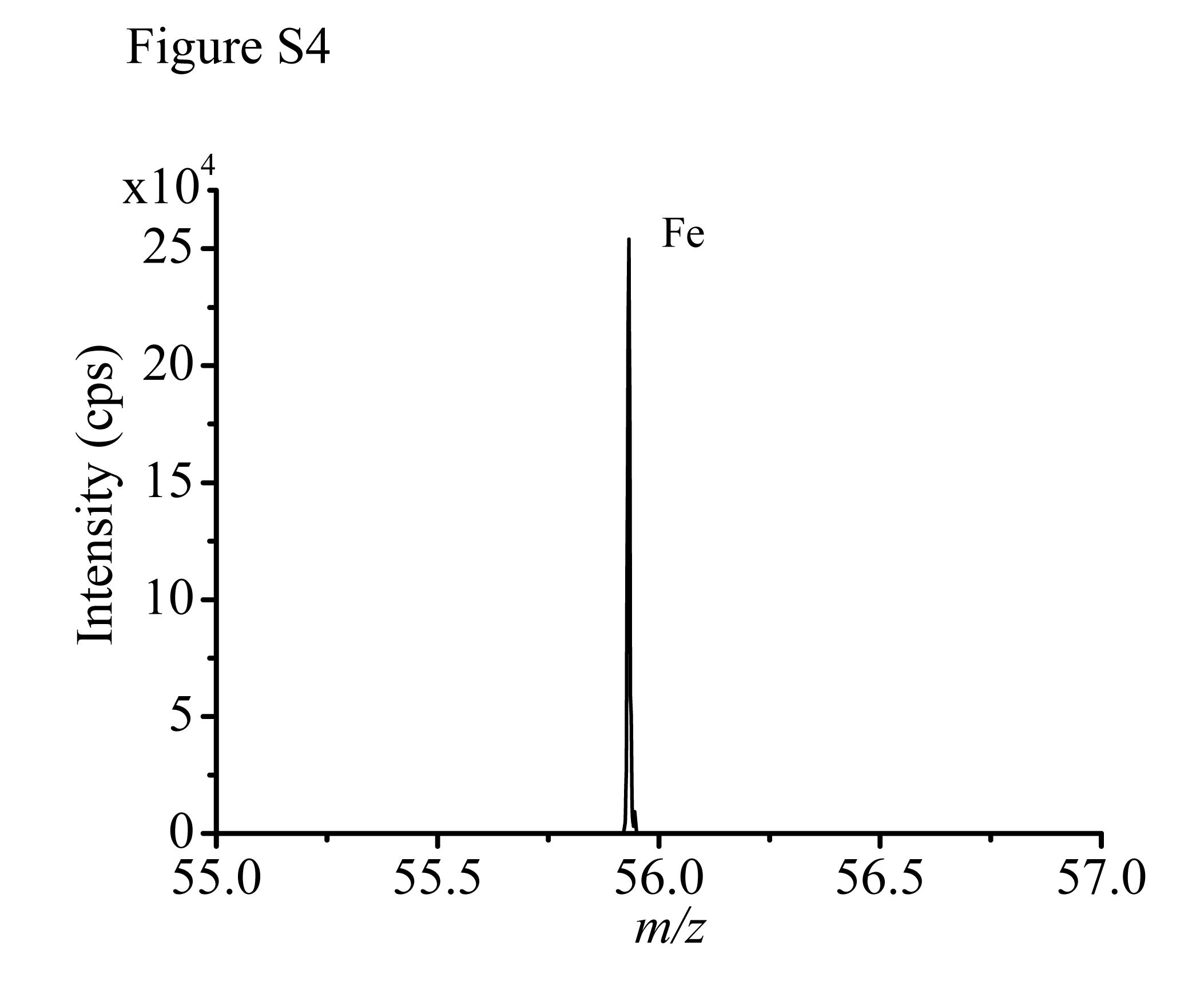

Supplement: Figure S4 — Identification of Fe in purified PduQ-His6 using ICP-MS (cps: counts per second). (TIF) [file pone.0047144.s004.tif]

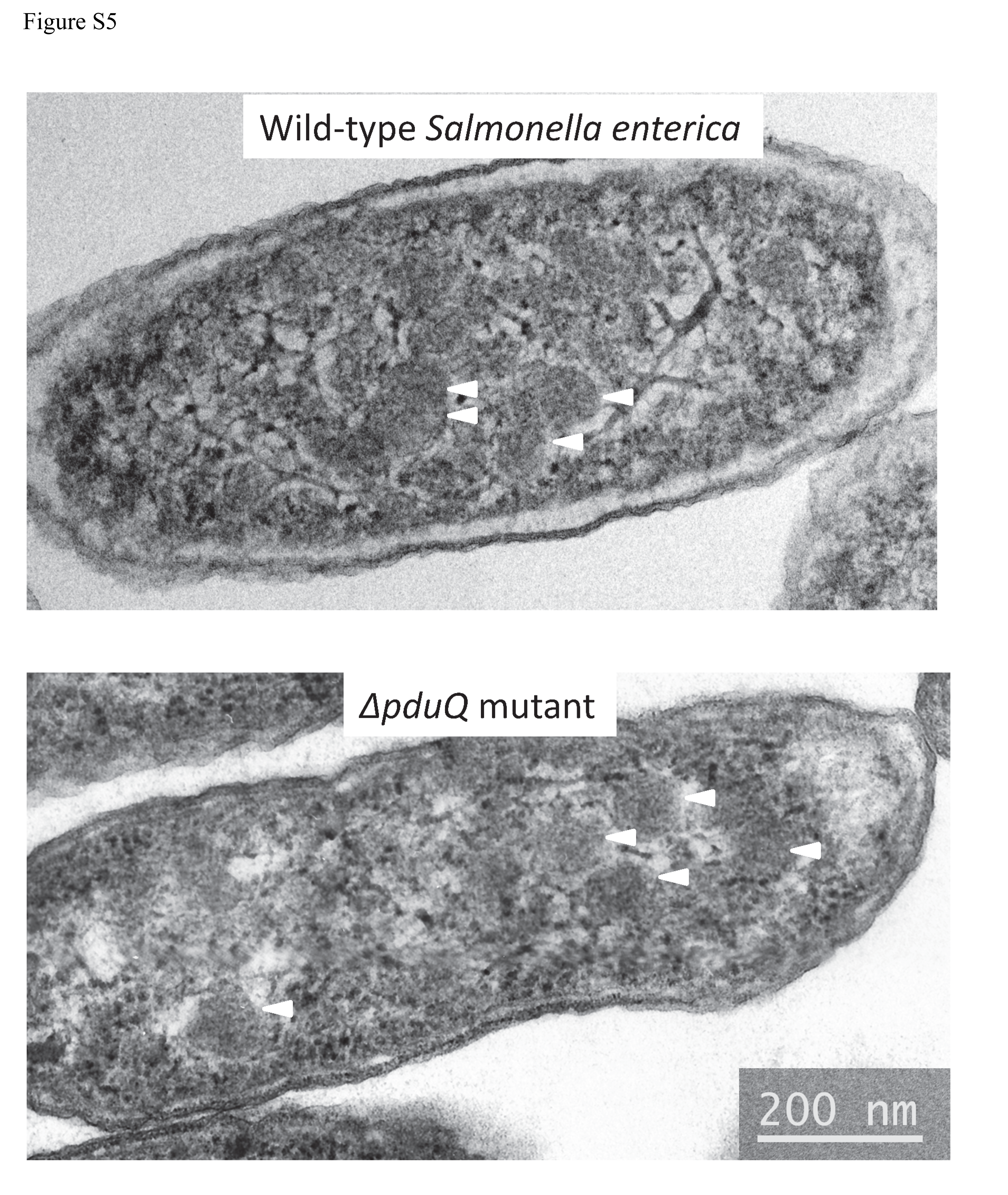

Supplement: Figure S5 — Electron microscopy of wild-type Salmonella enterica and a pduQ deletion mutant. Triangles point to microcompartments. A number of sections were examined and the cells shown are representative. (TIF) [file pone.0047144.s005.tif]

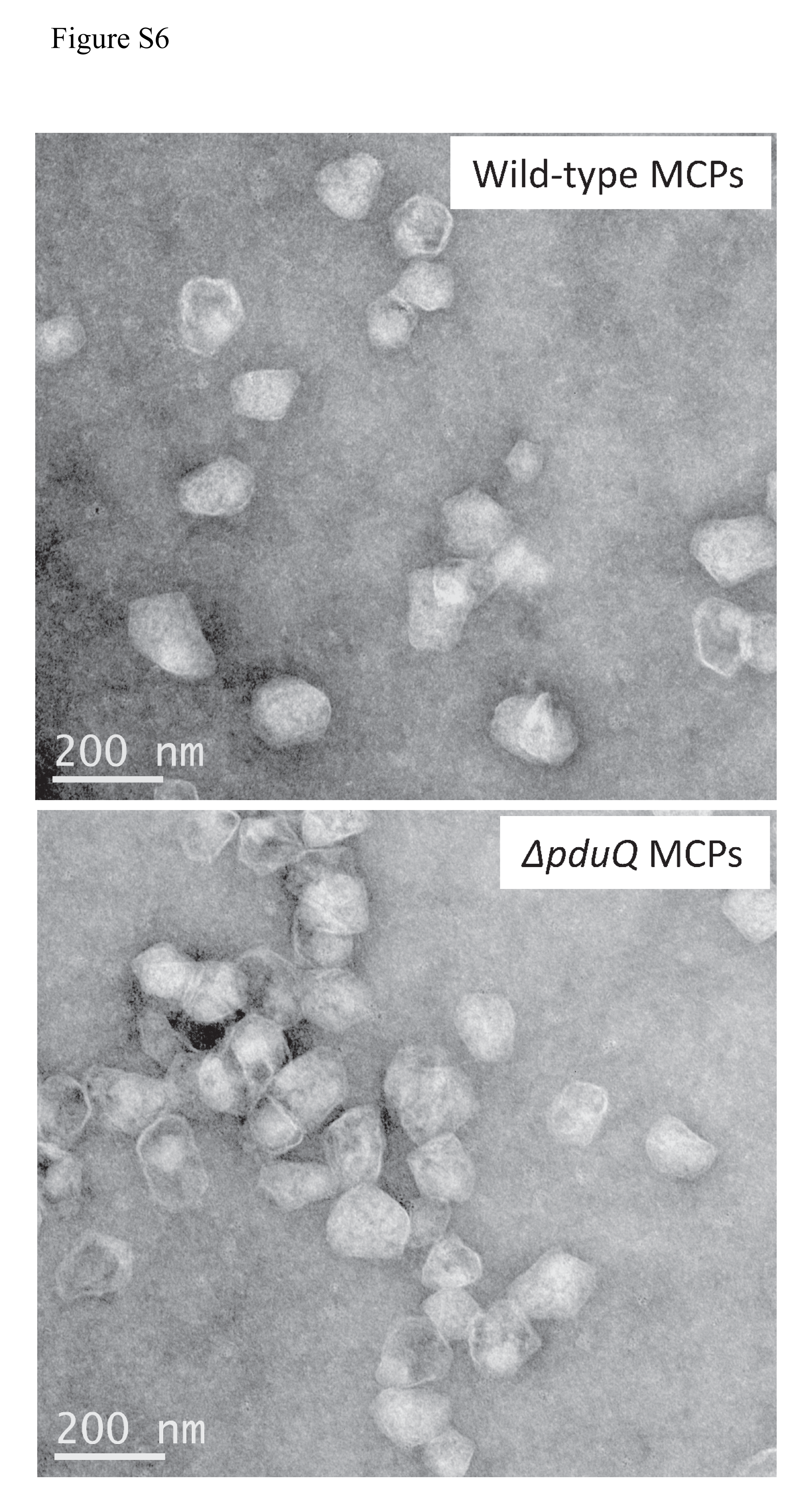

Supplement: Figure S6 — Electron microscopy of MCPs purified from wild-type Salmonella enterica and a pduQ deletion mutant. The image shown is a representative negative stain. (TIF) [file pone.0047144.s006.tif]
